# 2557. Development of Rabbit Models of Ventilator-Associated Carbapenem-Resistant *Acinetobacter baumannii* Pneumonia

**DOI:** 10.1093/ofid/ofad500.2174

**Published:** 2023-11-27

**Authors:** Vidmantas Petraitis, Ruta Petraitiene, Ethan Naing

**Affiliations:** Weill Cornell Medicine of Cornell University, New York, New York; Weill Cornell Medicine, New York, New York; Transplantation–Oncology Infectious Disease Program, Translational Research Laboratory, Division of Infectious Diseases, Weill Cornell Medicine, New York, NY 10065, US, New York, NY

## Abstract

**Background:**

Carbapenem-resistant (CR) Gram-negative bacteria are emerging worldwide as global public health threats. Mortality rates from these pathogens are especially high in immunocompromised patients. Ventilator-associated bacterial pneumonia (VABP) is among the most intractable of CR Gram-negative bacterial infections. We therefore aimed to develop rabbit animal models of VABP caused by CR strains of *Acinetobacter baumannii.*

**Methods:**

Persistently neutropenic NZW rabbits were used throughout the study. A novel endotracheal tracheostomy system was developed for intubation. The early-phase intubated model (0-24h) received mechanical ventilation, while the late-phase intubated model (72-96h) was ambulatory. Ventilators were set to FiO_2_=40% and PEEP=8mmHg. The following outcome variables were assessed: survival, residual tissue bacterial burden (log CFU/g), residual BAL bacterial burden (log CFU/mL), lung weights, pulmonary lesion score, histology, pulse O_2_ saturation, radiographic imaging, pro-inflammatory cytokines, quantitative tissue and BAL PCR, and histology.

**Results:**

Within the first 12h post-inoculation, mean bacterial burdens in lung tissue and BAL fluid, respectively, which were established at approximately 10^6^ CFU/g and 10^5^ CFU/mL, persisted through 24h in the early-phase model and were increased in the late-phase model (72-96h) to approximately 10^7^ CFU/g, and 10^6^ CFU/mL. Mean Max SpO_2_ was ≥99 mmHg and mean nadir SpO_2_ was ≥74 mmHg. Serial thoracic radiographs over the course of 12-96h demonstrated progressive multilobar pneumonic infiltrates, while lung histology revealed progressive development of focal bronchopneumonia, intra-alveolar hemorrhage, alveolar epithelial cell necrosis, macrophage activation, monocytic infiltration, alveolar septal disruption, and microcolonies of bacteria.

**Conclusion:**

The new rabbit model of VABP produced by CR *A. baumannii* recapitulates the pathophysiological, microbiological, diagnostic imaging, and histological patterns of human disease by which to assess critically needed new antimicrobial agents against this lethal infection.

**Disclosures:**

**All Authors**: No reported disclosures

